# Cytogenetic characterization, rDNA mapping and quantification of the nuclear DNA content in *Seriolella violacea* Guichenot, 1848 (Perciformes, Centrolophidae)

**DOI:** 10.3897/CompCytogen.v14i3.53087

**Published:** 2020-07-14

**Authors:** Cristian Araya-Jaime, Claudio Palma-Rojas, Elisabeth Von Brand, Alfonso Silva

**Affiliations:** 1 Instituto de Investigación Multidisciplinar en Ciencia y Tecnología, Universidad de La Serena, Casilla 554, La Serena, Chile Universidad de La Serena La Serena Chile; 2 Laboratorio de Genética y Citogenética Vegetal, Departamento de Biología, Universidad de La Serena. La Serena, Chile Universidad Católica del Norte Sede Coquimbo Coquimbo Chile; 3 Departamento de Biología Marina Facultad de Ciencias del Mar, Universidad Católica del Norte Sede Coquimbo, Casilla 117, Coquimbo, Chile Universidad de La Serena La Serena Chile; 4 Laboratorio Cultivo de Peces, Facultad de Ciencias del Mar, Universidad Católica del Norte Sede Coquimbo, Casilla 117, Coquimbo, Chile Universidad Católica del Norte Sede Coquimbo Coquimbo Chile

**Keywords:** chromosomal statis, CMA_3_ staining, genome size, Repetitive DNAs

## Abstract

*Seriolella violacea* Guichenot, 1848 is an important component of the fish fauna of the Chilean coast and is of great economic interest. Cytogenetic information for the family Centrolophidae is lacking and the genomic size of five of the twenty-eight species described for this family are is barely known. This study aimed to describe for the first time the karyotype structure via classical and molecular cytogenetics analysis with the goal of identifying the constitutive heterochromatin distribution, chromosome organization of rDNA sequences and quantification of nuclear DNA content. The karyotype of *S. violacea* is composed of 48 chromosomes, with the presence of conspicuous blocks of heterochromatin on chromosomal pairs one and two. FISH assay with a 5S rDNA probe, revealed the presence of fluorescent markings on the heterochromatic block of pair one. The 18S rDNA sites are located exclusively on pair two, characterizing this pair as the carrier of the NOR. Finally, the genomic size of *S. violacea* was estimated at 0.59 pg of DNA as C-value. This work represents the first effort to document the karyotype structure and physical organization of the rDNA sequences in the *Seriolella* genome, contributing with new information to improve our understanding of chromosomal evolution and genomic organization in marine perciforms.

## Introduction

In recent years fish cytogenetics has accumulated data that establish evolutionary trends, phylogenetic relationships among different families, species and populations (Arai 2011). This information is of great importance for the management and conservation of natural stocks (Carvalho-Costa et al. 2008). Currently the karyotypes of only 2% of all global marine fish are known (Galetti et al. 2000; Vega et al. 2002; Arai 2011). These studies have been focusing on just a few families of reef and pelagic fish, such as Gerreidae (Calado et al. 2013), Scombridae (Soares et al. 2013), Gobiidae (Lima-Filho et al. 2012), Labridae (Molina et al. 2012; Paim et al. 2014), Haemulidae (Nirchio et al. 2007; Neto et al. 2011) Carangidae (Chai et al. 2009) and Rachycentridae (Jacobina et al. 2011) preferably distributed in the Atlantic Ocean. According to Jara-Seguel et al. (2011) the marine fish fauna of Chile has been little studied, with known cytogenetic data for only some species of the Atherinidae, Galaxiidae, Kyphosidae, Mugilidae, Ophidae and Paralichthydae families being available.

*Seriolella violacea* (Guichenot, 1848) is an important component of the fish fauna of the Chilean coast and has great economic value (Ojeda et al. 2000). This species has an epipelagic gregarious behavior, forming schools near the coast; adults are found in areas of the continental shelf, as well as within protected bays, along the entire northern coast of Chile. Due to their rapid growth, adaptability and potential market, they currently represent an important candidate for the start of cultivation programs (Angel and Ojeda 2001; Navarrete et al. 2014).

No cytogenetic information is available for the family Centrolophidae, and the chromosomal constitution of the 28 species described in this family is unknown (Arai 2011). In addition the genomic size of five species (Hardie and Hebert 2004) is barely known. Due to this lack of biological information and the high potential for aquaculture that these species represent, it is essential to carry out a cytogenetic characterization; the karyotype and genome size are two primary genetic characteristics of the species, which are of great importance, when studying taxonomy, phylogenetic relationships, evolution and molecular biology.

Considering the absence of cytogenetic information on the Centrolophidae and the biological and economic importance of these pelagic fish, this study aims to describe for the first time the karyotype structure using classical and molecular cytogenetics analysis and quantification of nuclear DNA content in *Seriolella violacea*.

## Material and methods

Six individuals, four males and two females, of *S. violacea* were obtained from the Laboratorio Central de Cultivos Marinos belonging to the Universidad Católica del Norte, Coquimbo-Chile. Mitotic chromosomes were obtained from cell suspensions of the anterior kidney, following the protocol established by Foresti et al. (1993). Approximately 20 metaphase spreads from different individuals were analyzed to confirm the diploid number and karyotype structure of *S. violacea*. The C-banding was carried out according to Sumner (1972); and the use of GC-specific fluorochrome Chromomycin A_3_(CMA_3_) following Schweizer (1976). The chromosomes were classified according to Levan et al. (1964).

The 18S rDNA and the 5S rDNA probes were obtained by PCR (Polymerase Chain Reaction) from genomic DNA of *Seriolella violacea* using primers NS1F(5’-GTAGTCATATGCTTGTCTC-3’), and NS8R(5’-TCCGCAGGTTCACCTACGGA-3’) (Cioffi et al. 2009) and 5SA (5’- TACGCCCGATCTCGTCCGATC-3’) and 5SB (5’-GCTGGTATGGCCGTAGC-3’) (Pendás et al. 1994), respectively, and subsequently labeled with biotin-16-dUTP and digoxigenin-11-dUTP.

FISH was performed under high stringency conditions using the method described by Pinkel et al. (1986). Slides were incubated with RNase (50 μg/ml) for 1 h at 37 °C. Then the chromosomal DNA was denatured in 70% formamide/2× SSC for 5 min at 70 °C. For each slide, 30 μl of hybridization solution was denatured for 10 min at 95 °C, dropped on the slides and hybridized overnight at 37 °C in a 2× SSC moist chamber. Probe detection was carried out with Avidin-FITC (Sigma) or anti-digoxigenin-rhodamine (Roche). Chromosomes were counterstained with DAPI (4’,6-diamidino-2-phenylindole, Vector Laboratories).

Measurements of nuclear DNA content (C-value) were done by microdensitometry in erythrocytes obtained from adult specimens (2♀ and 2♂), analyzing 200 nuclei per sample, using the software Image Pro-Plus 4.0. (Media Cybernetics). The blood was dispersed on slides, air dried, fixed in methanol-acetic acid (3:1 v/v) at 4 °C for 24 h and stained with the Feulgen reaction (Jara-Seguel et al, 2008). Nuclear optic density (OD) is calculated by the software according to the formula OD = log10(1/T) = – log10T; where T = intensity of transmitted light/intensity of incident light. From this estimation, the computer integrates the values of OD obtained for each one of the pixels and it calculates the integrated optical density (IOD = ƩOD). The IOD values, in arbitrary units, were converted to absolute mass of DNA by comparison with erythrocyte smears of rainbow trout (*Oncorhynchus mykiss* (Walbaum, 1792), 2C = 5.5 pg, 2n = 58–60) (Hartley and Horne 1985).

## Results

**Figure 1.**
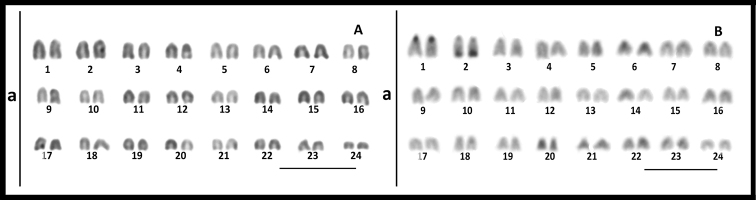
Conventional Giemsa-stained (**A**) and C-banding (**B**) in *Seriolella violacea*. Scale bar: 10 µm.

**Figure 2.**
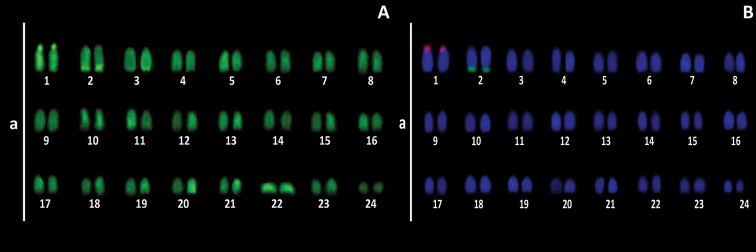
Karyotypes of *Seriolella violacea* after CMA_3_ staining (**A**) and dual color FISH with 18S rDNA (green) and 5S rDNA (red) probes (**B**). Scale bar: 10 µm.

The karyotype of *S. violacea* shows 24 pairs of chromosomes (2n = 48; FN = 48), all acrocentric (Fig. 1A). No morphologically differentiated sex chromosomes were found when metaphase plates from males and females were compared. C-positive blocks of constitutive heterochromatin (HC) were observed in pericentromeric regions of few chromosomes, highlighting the presence of two conspicuous HC blocks, one of them in the pericentromeric region of pair one, while the other was in the telomeric region of pair two (Fig. 1B). In addition, these two conspicuous blocks were positive for chromomycin A_3_ staining (Fig. 2A).

Dual FISH detected 18S and 5S rDNA probes on different chromosome pairs (Fig. 2B). Mapping the 5S rDNA probe revealed the presence of fluorescent markings on the heterochromatic block of pair one. The 18S rDNA sites are located exclusively on pair two, in a position coincident to heterocromatics/CMA_3_ positive blocks, characterizing pair two as the pair carrying the NOR.

Finally, the nuclear DNA content measured in erythrocytes of *S. violacea* was estimated to be 1.18 ± 0.04 pg (average IOD = 14345 arbitrary units), with a coefficient of variation of 4.2%. Since *S. violacea* is a diploid organism (2n = 48, n = 24), the C-value of 0.59 pg of DNA (Table 1), is equivalent to 578.2 megabase pairs (Mbp).

**Table 1.** Known genomic sizes C-Value(pg) for representatives of the Centrolophidae family.

Species	C-Value	Method	Cell Type	St. Species	Reference
*Centrolophus niger*	0.70	FIA	RBC	BS, GD, OM, RP	Hardie and Hebert 2004
*Hyperoglyphe antarctica*	0.77	FIA	RBC	BS, GD, OM, RP	Hardie and Hebert 2004
*Schedophilus huttoni*	0.76	FIA	RBC	BS, GD, OM, RP	Hardie and Hebert 2004
*Seriolella punctata*	0.78	FIA	RBC	BS, GD, OM, RP	Hardie and Hebert 2004
*Tubbia tasmanica*	0.76	FIA	RBC	BS, GD, OM, RP	Hardie and Hebert 2004
*Seriolella violacea*	0.59	FIA	RBC	GD, OM	in this work

**FIA**: Feulgen Imagen Analysis, **RBC**: Red Bloods Cells, **BS**: *Betta splendens*, **GD**: *Gallus domesticus*, **OM**: *Oncorhynchus mykiss*, **RP**: *Rana pipens*.

## Discussion

There are no data related to the organization of the repetitive fraction of the genome in the family Centrolophidae. Nevertheless, studies within the marine perciform order, specifically in representatives of the families Ephippidae, Serranidae, Lutjanidae, and Haemulidae have permitted the recognition of a diploid number of 48 chromosomes (completely acrocentric); the non-syntenic state of sequences 5S rDNA and 18S rDNA; and the presence of a single NOR, establishing this pattern as a plesiomorphic characteristic for marine perciforms (Chai et al. 2009; Arai 2011; Neto et al. 2011; Costa et al. 2016; Paim et al. 2017). The repetitive fraction of the genome can be a useful tool for identifying recent genomic changes that have occurred during the evolutionary process, as well as act as potential hotspots for chromosomal rearrangements (Ozouf-Costaz et al. 2004; Valente et al. 2011; Yano et al. 2014). In this sense, *S. violacea* presents exactly the cytogenetic pattern described for marine perciforms, highlighting the association of ribosomal clusters with heterochromatin blocks rich in CG bases in specific chromosome pairs. An association between 18S and 28S rDNA sequences and heterochromatin has been found in other fish, such as salmonids (Pendás et al. 1994; Fujiwara et al. 1998), species of the genera *Epinephelus* Bloch, 1793 (Sola et al. 2000), *Imparfinis* Eigenmann & Norris, 1900 and *Pimelodella* Eigenmann & Eigenmann, 1888 (Gouveia et al. 2013), *Orestias* Valenciennes, 1839 (Araya-Jaime et al. 2017) and sturgeon species (Fontana et al. 2003). This suggests that the repeated HC sequences play an important role and exercise diverse functions in the eukaryotic genome (Grewal and Jia 2007). It has even been postulated that heterochromatin is involved in maintaining the structure of the nucleolus and the integrity of ribosomal DNA repeats (McStay and Grummt 2008). Visualization of a single carrier pair sequence for 18S rDNA is one of the most common features observed in the fish genome, unlike what was observed for the gene 5S ribosomal which may present variations in the chromosomal distribution, apparently through its association with transposable elements, suggesting independent evolutionary pathways for both types of rDNA (Pendás et al. 1994; Martins and Galetti 2001; Cabral-de-Mello et al. 2011; Scacchetti et al. 2012; Sene et al. 2014; Santos et al. 2017; Usso et al. 2019). Teleosts exhibit low levels of compartmentalization in their genomes, which would suggest that the configuration in *S. violacea*, observed for the two types of ribosomal DNA, would represent a relatively simple to organization state (Medrano et al. 1988).

Finally, 0.59 pg of DNA (C-value) measured in erythrocytes of *S. violacea* represents a significantly (20%) lower nuclear DNA content than that of the five species of the Centrolophipadae family analized (Table 1), which on average reach 0.75 pg DNA. Thus, this value represents the smallest genome size known to the family. Currently there are data of nuclear DNA content for 634 species of Perciformes, estimating an average of 0.94 pg of DNA (C-value) for this order of fish, with minimum values of 0.39 pg in *Scienops ocellatus* (Linnaeus, 1766) and maximum of 2.60 in *Lagodon rhomboides* (Linnaeus, 1766) (Hardie and Hebert 2004; Gregory 2020). The evolutionary role genome size plays is the subject of much discussion, but computational biology has helped to model some patterns. These patterns are clearer when the nuclear DNA content is related to species life history attributes, especially with regards to effective population sizes and their gene flow rates, showing an inverse relationship between population size and the size of the genome (Vinogradov 2004; Labar and Adami 2017; Bobay and Ochman 2018).

## Conclusion

In this work, the karyotype of a representative of the Centrolophidae family, *S. violacea*, is described for the first time. Its karyotype is made up of 48 acrocentric chromosomes (2n = 48; FN = 48), simple NOR and ribosomal cistrons (5S-18S rDNA) are not synthetic. Meanwhile, the nuclear DNA content, C-value, was found to be 0.59 pg. It is necessary to perform additional studies physically mapping repetitive DNAs in the other representatives of the genus *Seriolella* Guichenot, 1848, in order to understand the involvement of these sequences in the process of chromosomal evolution that these fish may be experiencing. It is especially necessary to analyze the chromosomal microstructure, given the chromosomal stasis that most marine perciforms present, as this will also expand knowledge of fish fauna which is facing serious conservation issues.
